# EGFR, CD10 and proliferation marker Ki67 expression in ameloblastoma: possible role in local recurrence

**DOI:** 10.1186/1746-1596-7-14

**Published:** 2012-02-02

**Authors:** Azza Abdel-Aziz, Maha M Amin

**Affiliations:** 1 Pathology Department, Faculty of medicine, Mansoura University, Egypt

**Keywords:** Ameloblastoma, EGFR, CD10, Ki67 and recurrence

## Abstract

**Background:**

Ameloblastoma is an odontogenic neoplasm characterized by local invasiveness and tendency towards recurrence.

**Aims:**

Studying the role played by EGFR, CD10 and Ki67 in the recurrence of ameloblastoma.

**Methods:**

This study was carried out on 22 retrospective cases of mandibular ameloblastoma from the period from Jan 2002 to Jan 2008 with follow up period until Jan 2011 (3 to 8 years follow up peroid). Archival materials were obtained from pathology department, Mansoura university. Paraffin sections of tumor tissue from all cases were submitted for routine H&E stains and immunohistochemistry using EGFR, CD10 and Ki67 monoclonal antibodies. Statistical analysis using of clinical data for all patients, tumor type, EGFR, CD10 and Ki67 expression in relation to recurrence were evaluated.

**Results:**

Among the 22 cases, 10 cases were males and 12 were females with sex ratio 1:1.2. Age ranged from 34 to 59 years old with a mean age 44.18 year. Five cases showed local recurrence within studied period and proved by biopsy. No statistically significant relation was found between local recurrence and patient age, tumor size, tumor type, EGFR expression. There was a significant relation between CD10 expression as well as Ki67 labelling index and recurrence (P value = 0.003, 0.000 respectively).

**Conclusion:**

Evaluation of CD10 and Ki67 status together with conventional histological evaluation can help in providing more information about the biologic behavior of the tumor, while EGFR could be a target of an expanding class of anticancer therapies.

Since ameloblastomas are EGFR-positive tumors, anti-EGFR agents could be considered to reduce the size of large tumors and to treat unresectable tumors that are in close proximity to vital structures.

**Virtual Slides:**

The virtual slide(s) for this article can be found here:

http://www.diagnosticpathology.diagnomx.eu/vs/1902106905645651

## Introduction

Ameloblastoma is the most common odontogenic neoplasm that accounts for about 1% of all oral tumors [1] and arises from the epithelium of the dental lamina affecting mainly the posterior mandible (80%) and to a lesser extent the posterior maxilla (20%). It usually affects adults in the 4th - 5th decades of life [2]. Ameloblastoma is generally benign, grows slowly and is not associated with symptoms until it becomes large. However, it is locally aggressive and displays a strong tendency to recur especially if not adequately removed [3,4] and even metastasize in rare conditions[5]. It is important to assess the type according to recent WHO classification (solid/multicystic, unicystic, desmoplastic and peripheral), localization, and size of the tumors as well as age of the patient [6]. There are two basic histopathologic patterns; the follicular and plexiform without clinical relevance [7].

The epidermal growth factor receptor (EGFR) is a transmembrane receptor tyrosine kinase comprising an extracellular ligand binding domain, a transmembrane domain, and an intracellular tyrosine kinase domain [8]. Binding of epidermal growth factor results in EGFR dimerization and subsequent activation of the intrinsic tyrosine kinase activity. Phosphorylated EGFR concomitantly triggers downstream mitogenic signaling via both the MAPK and PI3K pathways [9].

Expression of epidermal growth factor receptor (EGFR) regulates proliferation of both normal and neoplastic cells [10,11]. It has been observed in normal epithelia, including the oral mucosa, and might provide epigenetic control of odontogenesis [12].

CD10 (common acute lymphoblastic leukemia antigen, CALLA) is a 100-kDa transmembrane glycoprotein, also known as neutral endopeptidase (NEP), involved in the cleavage and inactivation of certain peptide hormones important for signal transduction [13]. CD10 is expressed by a variety of normal cell types, including lymphoid precursor cells, germinal center B lymphocytes and some epithelial cells as gastric mucosa [14]. First, CD10 was reported in relation to lymphoid neoplasms. However, its expression is also reported in malignant epithelial neoplasm and melanoma [15]. Although CD10 expression is observed in neoplastic cells, there are reports of its expression in stromal cells. Moreover, there are cumulative data indicating that CD10 expression by stromal cells is involved in carcinogenesis and it is supposed to be a novel prognostic factor in some malignant tumors [14].

Identification of proliferating activities in tumors may be useful to predict their biological behavior. Ki-67 protein is a nuclear non-histone protein which is required for maintaining the cell cycle. Ki-67 is expressed by proliferating cells in all phases of the active cell cycle (G1, S, G2 and M phase) but is absent in resting (G0) cells [16]. Ki-67 has been used to determine the proliferation rate of many tumors, including ameloblastomas [17].

For a better understanding of the aggressive behavior of ameloblastomas, their expression of growth factor receptors, metalloproteinases and their proliferative activity have been investigated using immunohistochemical methods. So, the aim of the present study is evaluation of EGFR, CD10 expression and Ki-67 labelled index in ameloblastoma and their relation to recurrence.

## Materials and methods

This retrospective study was carried out on mandibular ameloblastoma specimens received in the pathology department from the period from Jan 2002 to Jan 2008. Follow up data were retrieved from patient's files for at least 3 years duration. Each specimen was coded and patient's name was not shown for ethical reasons. Age of the patients, sex, tumor size, site and recurrence were revised. 

Ethical approval of this study was not required by our institution as this study was based on retrospective analysis dealing with archival paraffin slides and blocks, not related to patient's privacy, impairment or treatment.

### Histopathology

Sections of 4 um thickness have been cut from formalin fixed paraffin embedded blocks of archival ameloblastoma tissues for routine H&E, others were prepared on charged slides for immunohistochemistry. Examination of three tumor slides from each specimen were done on an Olympus CX31 light microscope. Pictures were obtained by a PC-driven digital camera (Olympus E-620). The computer software (Cell*, Olympus Soft Imaging Solution GmbH) allowed morphometric analysis to be performed.

### Immunohistochemistry

Immunohistochemical analysis for EGFR, CD10 and KI67 with a labelled streptavidin- biotin-peroxidase complex technique was performed on tumor sections. The antibodies used were monoclonal antibody against EGFR (clone H11-DAKO, DakoCytomation, Carpinteria, CA, USA), at 1:25 dilution, CD10 (Santa Cruz Biotechnology Inc., sc-19993, dilution 1: 50) and Ki-67 (clone MIB-1, N1633, Dako Corporation, Carpinteria, CA, USA, RTU). Detection kit used was high sensitive kit (DakoCytomation envision +dual link system peroxidase code K4061) using DAB as chromogene. EGFR immunostaining required antigen retrieval with 0.2% trypsin, CD10 and Ki67 immunostaining required pretreatment with 1 mM EDTA (at pH 8.0) for 20 minutes in microwave oven. Proper positive and negative controls were performed. Normal oral mucosa was used as positive control for EGFR, tonsils for Ki67 and CD10. As a negative control, sections were stained without the addition of a primary antibody.

### Immunohistochemical Analysis

As for the immunohistochemistry assessment, Slides were scanned by X40 magnification. Ten cellular areas selected (i.e. the so-called hot spots) and evaluated at X400 magnification by two pathologists.

#### Assessment of EGFR

Both membranous and cytoplasmic staining of EGFR were evaluated. The proportion of stained cells and staining intensity were combined to assess the immunohistochemical staining according to previous records [18-20]. Staining intensity was evaluated on a semi-quantitative three-point scale: 0--no staining, 1--weak and 2--strong staining. The final EGFR staining score was calculated by multiplying the percentage of positively stained tumor cells by the staining intensity. Accordingly, the highest score for a given tumor would be equal to 2.

#### Assessment of CD10

Stromal CD10 was scored according to similar system suggested by Iwaya et al, [21]. and Zu et al. [22]. as follow 0, equivalent to the negative control; 1, weak cytoplasmic stain; 2, moderate stain; 3, intense stain. The percentage of stained cells was also scored on a semi-quantitative 4-point scale as: 0, < 10%; 1, 10-25%; 2, 25-50%; 3, > 50%. Then, combining the score of staining intensity and percentage of stained cells: a score of 0-1 was -, 2 was +, 3-4 was ++ and 5-6 was +++.

#### Assessment of Ki67

Ki67 labeling index was done by calculating the ratio of positive nuclei in relation to total number of neoplastic nuclei in 10 HPFs. The labeling index (number of positive tumor cells/total number of tumor cells expressed as a percentage) was calculated in every specimen [23].

### Statistical analysis

All parameters included age, tumor size, pathologic type, EGFR, CD10 expression in stromal cells and ki67 labeling index with recurrence were evaluated by statistical analysis. The statistical analysis of data was done by using statistical package for social science (SPSS) program version 14. Descriptive statistics were done. The presented data was asymmetrical. One Way Anova test (for EGFR, Ki67) and Chi square test (for the remaining data including CD10) were performed to determine significance of the relations. Survival analysis of recurrence free survival was done by Kaplan-Meier analysis and log rank test was used for comparison between groups. Probability (p) values < 0.05 were considered significant.

## Results

This study was carried out on retrospective cases mandibular ameloblastoma received in the pathology department from the period from Jan. 2002 to Jan. 2008 with follow up period until Jan. 2011 (minimal 3 years follow up).

### Clinical characteristics

Ten cases were males and 12 were females with sex ratio 1:1.2. Age ranged from 34 to 59 years old with mean age 44.18 ± 6.97. Five cases (22.7%) showed local recurrence, four of them recurred 3-5 years after resection and one case recurred 2 years after

### Pathology results

**Figure 1:**
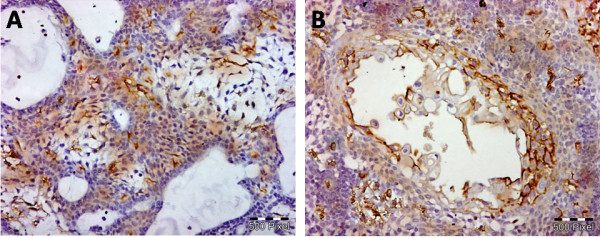
**EGFR immunostaining** (A) represents membranous and cytoplasmic EGFR expression in basal and stellate reticulum like cells. (B) represents membranous and cytoplasmic EGFR expression in focus of squamous differentiation (immunoperoxidase X200).

**Figure 2:**
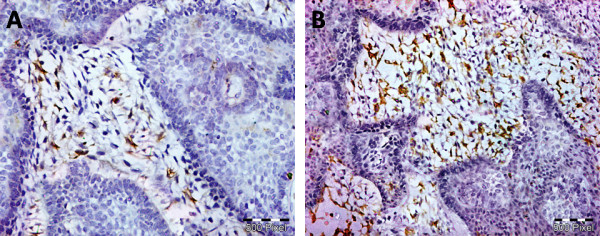
**Stromal CD10 immunostaining **(A) represents cytoplasmic and nuclear CD10 expression of mild intensity in about 15% of stromal cells. (B) represents CD10 expression of moderate to strong intensity in about 70% of stromal cells (immunoperoxidase X200).

**Figure 3:**
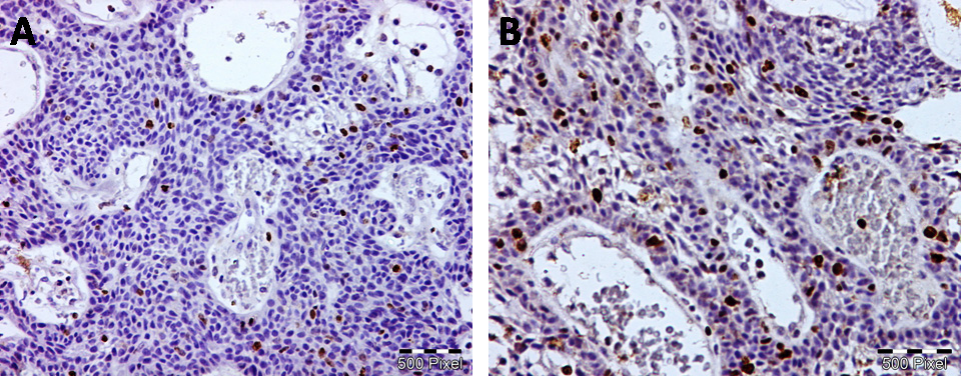
**Nuclear KI67 immunostaining** (A) represents Ki 67 labeling index reaching 7% of neoplastic cells. (B) represents Ki 67 index reaching 20% of neoplastic cells (immunoperoxidase X200).

Sixteen cases were follicular and six cases were plexiform subtype. As shown in table (1), all specimens demonstrated EGFR-positively stained tumor cells. Staining was membranous and cytoplasmic, both peripheral and central cells were stained (Figure 1). Two cases (9.1%) exhibited the maximum score of 2 and 20 (90.9%) scored between 0.2 and 1.8. Stromal CD10 immunostaining was negative in one case, 11 cases were (+), 7 cases were (++), 3 cases were (+++) (Figure 2). Regarding Ki67 labeling index, 17 cases showed low index with mean 8.29 ± 3.15 and 5 cases showed high index with mean 19 ± 2.12 (Figure 3).

**Table 1:** Summary of clinicopathologic finding of the studied cases.

*No*	*Age*	*Sex*	*Size (cm)*	*Pathological type*	*EGFR*	*CD 10*	*Ki67*	*Recurrence*
1	46	M	5	Plexiform	0.4	+	9	-

2	37	F	4	Plexiform	0.9	++	6	-

3	34	M	4	Tubular	1.6	+	6	-

4	38	M	3	Tubular	1	++	17	+

5	48	F	4	Plexiform	2	+++	20	+

6	42	M	5	Plexiform	1.6	+	5	-

7	48	M	3	Plexiform	1.2	+	7	-

8	52	F	6	Tubular	0.2	++	18	+

9	47	M	4	Plexiform	0.5	++	4	-

10	45	F	6	Plexiform	1.3	++	16	-

11	46	M	5	Plexiform	0.3	+++	22	+

12	45	F	6	Plexiform	1.5	+++	19	+

13	59	M	3	Tubular	1.8	-	11	-

14	35	F	4	Plexiform	1	++	9	-

15	43	F	3	Tubular	1.5	+	10	-

16	39	F	4	Plexiform	0.8	+	6	-

17	49	M	5	Plexiform	2	+	6	-

18	36	F	2	Plexiform	1.2	+	7	-

19	59	M	4	Tubular	0.2	+	9	-

20	46	F	5	Plexiform	1.4	+	14	-

21	42	F	2	Plexiform	2	+	7	-

22	36	F	4	Plexiform	0.6	++	9	-

Table (2) showed no statistically significant association between local recurrence and patient age (p = 0.774), tumor size (p = 0.375), tumor types (p = 0.467), and EGFR score (p = 0.774). There was a statistically significant relation between stromal CD10 and recurrence (P = 0.003); the stronger expression is associated with recurrence. Statistically significant relation was found between Ki67 labeling index and recurrence (P < 0.001); cases with recurrent ameloblastoma showed higher index (mean 19 ± 2.12) in contrast to non-recurrent cases (mean 8.29 ± 3.15).

**Table 2:** Demographic and pathologic criteria of studied cases in relation to recurrence.

		Non recurrent Cases(No)	Recurrent Cases(No)	***P Value***
Age groups (years)		6	1	0.774
	30-40	9	3	
	40-50	2	1	
	50-60			

Tumor size (cms)	< 2	5	1	0.375
	2-5	7	1	
	5-7	4	2	
	7-9	1	1	

Histological type	*Follicular Plexiform*	13	3	0.467
		4	2	

EGFR Expression	*No*	17	5	0.775
	*Mean*	1.07 ± 0.56	0.98 ± 0.70	
	*Median*	1	1	
	*Range*	0.2-2(1.8)	0.1-2(1.9)	

CD10 Expression	*0*	1	0	0.003
	*+*	11	0	
	*++*	5	2	
	*+++*	0	3	

Ki67 Index	*No*	17	5	0.000
	*Mean*	8.29 ± 3.15	19.00 ± 2.12	
	*Median*	7		
	*Range*	4-16(12)	19	
			17-22(18)	

**Figure 4:**
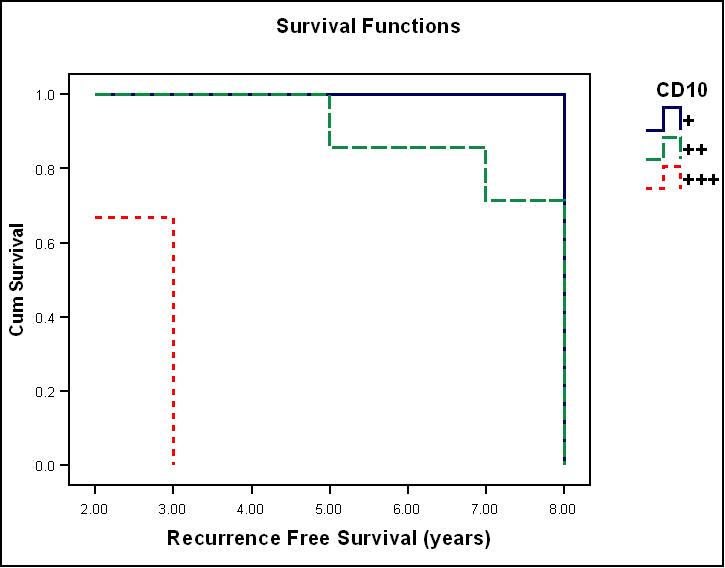
**Kaplan-Meier survival analysis for recurrence free survival (years) in relation to stromal CD10**. (One case with negative stromal CD10 was excluded from the curve).

**Figure 5:**
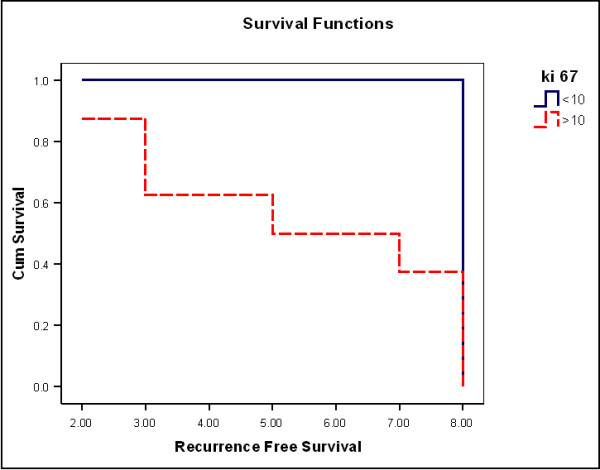
**Kaplan-Meier survival analysis for recurrence free survival (years) in relation to Ki67 labeling index**.

During the follow-up period, three cases having short recurrence free survival (RFS) (2-3 years) showed marked stromal CD10 expression (+++) and higher Ki67 labeling indices (19-22). On the other hand, cases with longer RFS (5, 7 years) showed moderate stromal CD10 expression (++) and less Ki67 labeling indices (17, 18). A statistically significant decrease in Patient's RFS was associated with stromal CD10 expression (P < 0.001 by Log-Rank test). The mean RFS was 8 ± 0.1 in cases showing mild stromal CD10 expression, 7.4 ± 0.43 in cases showing moderate stromal CD10 expression, 2.7 ± 0.33 in cases showing mild stromal CD10 expression (Figure 4). A statistically significant decrease in Patient's RFS was associated with Ki67 labeling index (P < 0.001 by Log-Rank test). The mean RFS was 8 ± 0.1 in cases with Ki67 index < 10, 5.1 ± 0.96 in cases with Ki67 index > 10 (Figure 5).

## Discussion

Ameloblastomas are locally invasive and destructive benign odontogenic tumors that arise from rests of the dental lamina. Their recurrence rate is high [3,4] even for patients that undergo surgical excision of the tumor even in excised tumors with free safety margin. The immunohistochemistry can describe the biological differences of these tumor types [24]. This study was carried out to elucidate the relationship between the expression of EGFR, CD10 and Ki-67 labelled index and ameloblastoma recurrence using clinical and pathological data.

Normal EGFR signaling plays an essential role in organ development, repair and in the regulation of cell survival. Aberrant signaling can be the result of EGFR overexpression by EGFR gene amplification or mutations with ligand-independent tyrosine kinase activity which could result in uncontrolled cell division; a predisposition for cancer [25,26]. EGFR upregulation appeared to be selectively expressed in a number of tumors as glioblastomas and lung cancer [27].

In the current study, all ameloblastomas exhibited EGFR immunoexpression with no identified relation to recurrence. Previous studies in the literature evaluated EGFR expression in ameloblastomas [19,28,29], and their results were divergent. Shrestha et al. [28] claimed that of the 23 cases of examined solid ameloblastomas, none demonstrated EGFR expression. However, Li et al. [29] reported that EGFR was detected in all six of their cases of ameloblastoma. Ueno et al., [30] examined 39 cases of solid ameloblastoma and EGFR expression was found in 30 (88%). Vered et al., [19] reported that all specimens were EGFR-positive using membranous, or both membranous and cytoplasmic staining as positivity criteria. Our results were mainly consistent with the latter finding. The differences between previous reports may be explained by the different positivity criteria adopted, such as only membrane labeling [28], or membrane and cytoplasmic labeling associated with labeling intensity [19,29].

CD10 is associated with differentiation and growth of neoplastic cells, and its expression is found to be increased with the increase of tumor dysplasia. O*g*awa et al., [31] found that there was no expression of CD10 in the stromal cells of normal colorectal tissue, while the percentage of CD10+ stromal cells were increasing adjacent to tumor cells with increasing dysplasia in adenomas and maximally found in stroma adjacent to invasive carcinoma [32]. Iwaya et al., [19] found that there was no staining in the stromal cells of noninvasive ductal carcinoma or normal breast tissue, while the frequency of positive stromal staining increased in cases with axillary lymph node metastases [21]. In the study of Makretzov et al., [32] stromal CD10 positivity, seen at the invasive front, was associated with higher tumor grade, and decreased survival in breast carcinoma, suggesting tumor-stromal interactions. In a study of CD10 in oral squamous cell carcinoma, it was found that CD10 positivity in stromal cells was an indicator of worse prognosis; a significant correlation was found with lymph node metastases, local recurrences, and histologic grade [33].

Ameloblastoma is a tumor that shows heterogeneous expression of CD10 [34]. Most recurrent tumors strongly express CD10 and could be a marker for aggressive behavior. Our data demonstrated that patients with tumors strongly express CD10 in the peritumoral stromal cells were more prone to local recurrence after resection with uninvolved cut margins. This is similar to the results in studies done by Lezzi et al., [35] and Masloub et al., [36]. It is possible then that the function of CD10 is employed primarily in invasion of extracellular matrix. The presence of CD10+ stromal cells may signify the aggressiveness of tumor. Similarly, Bilalovic et al., [37] reported metastatic behavior of melanoma cases with peritumoral CD10 positive stain.

Assessment of cell proliferation in many types of tumors is important together with histologically based tumor classification and has potential relevance as an indicator of tumor behavior, treatment response and relapse [38]. The results of this study showed that cellular proliferative activity as assessed by Ki67 labeling indices varied within recurrent and non-recurrent cases of ameloblastoma. There was a significant relation between labeling index of nuclear proliferation marker ki67 and recurrence of ameloblastoma. This is in concordance with Hirayama et al., [39] who found a high proliferative activity in recurrent ameloblastoma. In this study, the assessment of the cellular proliferation marker was shown to be reliable and reproducible.

All the above-mentioned immunohistochemical data indicated that the immunoexpression of CD10 and Ki67 labeling index may be good predictor for recurrence in ameloblastoma.

## Conclusion

Our data demonstrated that CD10-positive tumors with high Ki67 index were associated with high recurrence rate, while EGFR expression was not predictive for prognosis in ameloblastomas but may render such tumors candidate for the new targeted anti-EGFR treatment modalities. Moreover, we hope formulation of a target therapy against CD10 positive cells, including monoclonal antibody mediated-delivery of chemotherapy. Clinical surveys with larger study cohorts will be needed to verify our findings.

## Competing interests

The authors declare that they have no competing interests.

## Authors' contributions

AA contributed to study design, collecting data, analysis, writing the manuscript, performed statistical analysis and in deciding to submit the manuscript for publication. MMA contributed to revision and approval of final and revised manuscript draft, and in deciding to submit the manuscript for publication. All authors read and approved the final manuscript.
